# Neoadjuvant therapy for locally advanced gastric cancer patients. A population pharmacodynamic modeling

**DOI:** 10.1371/journal.pone.0215970

**Published:** 2019-05-09

**Authors:** Patricia Martin-Romano, Belén P. Solans, David Cano, Jose Carlos Subtil, Ana Chopitea, Leire Arbea, Maria Dolores Lozano, Eduardo Castanon, Iosune Baraibar, Diego Salas, Jose Luis Hernandez-Lizoain, Iñaki F. Trocóniz, Javier Rodriguez

**Affiliations:** 1 Department of Oncology, Clínica Universidad de Navarra, Pamplona, Spain; 2 Department of Pharmacy and Pharmaceutical Technology, School of Pharmacy, Universidad de Navarra, Pamplona, Spain; 3 Department of Radiology, Clinica Universidad de Navarra, Pamplona, Spain; 4 Department of Gastroenterology, Clínica Universidad de Navarra, Pamplona, Spain; 5 Department of Pathology, Clínica Universidad de Navarra, Pamplona, Spain; 6 Department of Surgical Oncology, Clínica Universidad de Navarra, Pamplona, Spain; University of South Alabama Mitchell Cancer Institute, UNITED STATES

## Abstract

**Background:**

Perioperative chemotherapy (CT) or neoadjuvant chemoradiotherapy (CRT) in patients with locally advanced gastric (GC) or gastroesophageal junction cancer (GEJC) has been shown to improve survival compared to an exclusive surgical approach. However, most patients retain a poor prognosis due to important relapse rates. Population pharmacokinetic-pharmacodynamic (PK/PD) modeling may allow identifying at risk-patients. We aimed to develop a mechanistic PK/PD model to characterize the relationship between the type of neoadjuvant therapy, histopathologic response and survival times in locally advanced GC and GEJC patients.

**Methods:**

Patients with locally advanced GC and GEJC treated with neoadjuvant CT with or without preoperative CRT were analyzed. Clinical response was assessed by CT-scan and EUS. Pathologic response was defined as a reduction on pTNM stage compared to baseline cTNM. Metastasis development risk and overall survival (OS) were described using the population approach with NONMEM 7.3. Model evaluation was performed through predictive checks.

**Results:**

A low correlation was observed between clinical and pathologic TNM stage for both T (R = 0.32) and N (R = 0.19) categories. A low correlation between clinical and pathologic response was noticed (R = -0.29). The OS model adequately described the observed survival rates. Disease recurrence, cTNM stage ≥3 and linitis plastica absence, were correlated to a higher risk of death.

**Conclusion:**

Our model adequately described clinical response profiles, though pathologic response could not be predicted. Although the risk of disease recurrence and survival were linked, the identification of alternative approaches aimed to tailor therapeutic strategies to the individual patient risk warrants further research.

## Introduction

Upper gastrointestinal tumors, including gastric cancer (GC) and gastroesophageal cancer junction (GEJC) remain a therapeutic challenge. Most patients are diagnosed with a locally advanced stage, defined as tumor growing through the gastric wall and/or regional lymph node involvement. To date, surgery with an extended lymphadenectomy and microscopically negative margins (R0) is the only potentially curative treatment [1, 2]. However, long-term survival of patients with a surgical exclusive approach remains low [3]. Multimodal strategies (MMS), including adjuvant chemotherapy (ChT) or chemoradiotherapy (CRT), perioperative chemotherapy and neoadjuvant chemo-radiation have been correlated with improved survival times when compared to surgery alone [4–9].

Molecular predictive biomarkers able to reliably assess individual patients’ risk of relapse in patients with locally advanced GC/GEJC are currently lacking. One possible alternative to predict outcome is the development of population pharmacokinetic-pharmacodynamic (PK/PD) models. These models attempt to integrate into a single computational framework patient and treatment related information with the aim of describing potential links between prognostic factors and patients’ outcome, and assessing model-based metrics as “drivers” for response to treatment [10]. This association may not be detected by a simple assessment of correlation. The paradigm has been used previously in other types of tumors such as prostate and small cell lung cancer [10, 11]. Another potential application of this modeling approach is the identification of ‘at risk-patients’, in order to tailor therapeutic strategies on an individual basis [11].

In the present work, we aimed to develop a computational model to characterize the relationship between the type of neoadjuvant therapy used, the histopathologic response, and the risk of relapse and death in patients with locally advanced GC and GEJC.

## Material and methods

### Patient characteristics

Patients with clinical stage Ib-III GC and GEJC treated at our institution with preoperative therapy followed by surgery were included in this analysis.

Patients were considered for a neoadjuvant treatment if they were aged ≥18, had a locally advanced (cT2–4 and/or N+) gastric or gastroesophageal junction (GEJ) adenocarcinoma according to the TNM system (American Joint Committee on Cancer, 7th edition), a good performance status (0–1 according to Eastern Cooperative Oncology Group [ECOG]), and an adequate hematological, renal, and liver function. Patients were excluded if they had non-adenocarcinoma histology tumors, distant metastatic disease (M1), positive peritoneal cytology or peritoneal carcinomatosis, as were those considered unfit for the treatment protocol due to comorbidities.

Baseline patient evaluation included clinical examination, blood tests, upper gastrointestinal tract endoscopic ultrasound (EUS) with biopsy, and chest–abdominal computerized tomography-scan (CT-scan) to define the extent of the disease. Preoperative therapy consisted of ChT alone or induction ChT followed by concurrent CRT. A multidisciplinary panel decided the most suitable preoperative strategy based on patient profile: age, ECOG, comorbidities, and the clinical stage of the primary tumor.

This study was conducted with the approval from the institutional review board and ethics committee of the Clinica Universidad de Navarra. Written informed consent was obtained from all patients included in the study.

### Neoadjuvant strategy

All patients received ChT on an outpatient basis and underwent regular clinical follow-up consisting of physical examination, blood tests and toxicity profile monitoring.

For patients receiving CRT, the design of the radiation technique and the target volume were individualized taking into account the extent and location of the primary tumor and the involved regional lymph nodes. The clinical target volume included the gross tumor volume along with the entire stomach, and the draining loco-regional lymph nodes (perigastric, suprapancreatic, celiac, splenic hilar, porta hepatis, and pancreatoduodenal). The last 3 cm of the distal esophagus were included in patients with GEJC. Treatment planning followed International Commission on Radiation Units and Measurements recommendations. In general, three fields with 15-MV photons were employed to deliver 45 grays (Gy) over 5 weeks with conventional daily fractions of 1.8 Gy, 5 days per week. Seven coplanar, equally spaced beams were applied with a variable number of segments in IMRT plans. All patients received oral fluoropyrimidine-based concurrent chemotherapy. Patients underwent weekly clinical evaluations that included physical examination, blood test monitoring, and therapy-induced toxicity management. Acute toxicity for both ChT and CRT was assessed according to the “Common Terminology Criteria for Adverse Events” version 4.0.

### Clinical response assessment

At the completion of the neoadjuvant protocol, primary tumor response was evaluated by CT-scan and/or EUS. Baseline, on-therapy and pre-surgery CT-scans were retrospectively reviewed by a senior radiologist (DCR) to quantify tumor burden changes using RECIST criteria [12]. A decrease in the sum of diameters of target lesions of 30%, taking as reference the baseline sum diameters by CT scan was considered a clinical response. A decrease on circumferential and/or depth of tumor invasion by EUS was also considered a clinical response.

### Surgery

Surgery was scheduled 4 to 6 weeks after the completion of the neoadjuvant treatment. Location and extent of the primary tumor determined the type of surgery. A subtotal gastrectomy was performed in distal tumors. For proximal tumors, and those located in the gastroesophageal junction (GEJ), either a subtotal or total gastrectomy extended to distal esophagus or an Ivor-Lewis esophagogastrectomy was performed. Spleen preservation was performed. An attempt was made to perform a D2 nodal dissection in all patients.

### Pathologic response assessment

A gastrointestinal pathologist performed the pathologic examination of all the surgical specimens. TNM status was assessed postoperatively in accordance with the 7th edition of the TNM-AJCC cancer staging. Pathologic response was defined as a reduction in the pathologic T and/or N stage (ypTNM) compared to baseline clinical staging (cTNM). A complete pathologic response (pCR) was considered when no evidence of residual tumor was found in the surgical specimen (ypT0 ypN0).

### Data analysis

The population approach was applied for the analysis using NONMEM version 7.3 software (Icon Development Solutions, Ellicott City, MD, USA). The first order conditional estimation (FOCE) method with LAPLACIAN was used.

#### Model selection criteria

Model selection was based on the evaluation of different statistical and graphical criteria: (i) the minimum value of the objective function (MVOF) provided by NONMEM, which was equal to -2xLog likelihood (-2LL); -2LL differences of 3.86, 6.63, 7.88 or 11.87 were considered significant at the 5, 1, 0.5 and 0.1% levels, respectively, for nested models differing in one parameter; (ii) the precision of parameter estimates expressed as relative standard error in percentage [RSE(%)] using the information provided by NONMEM and (iii) visual inspection of the visual predictive checks.

#### Model development

**Fig 1:**
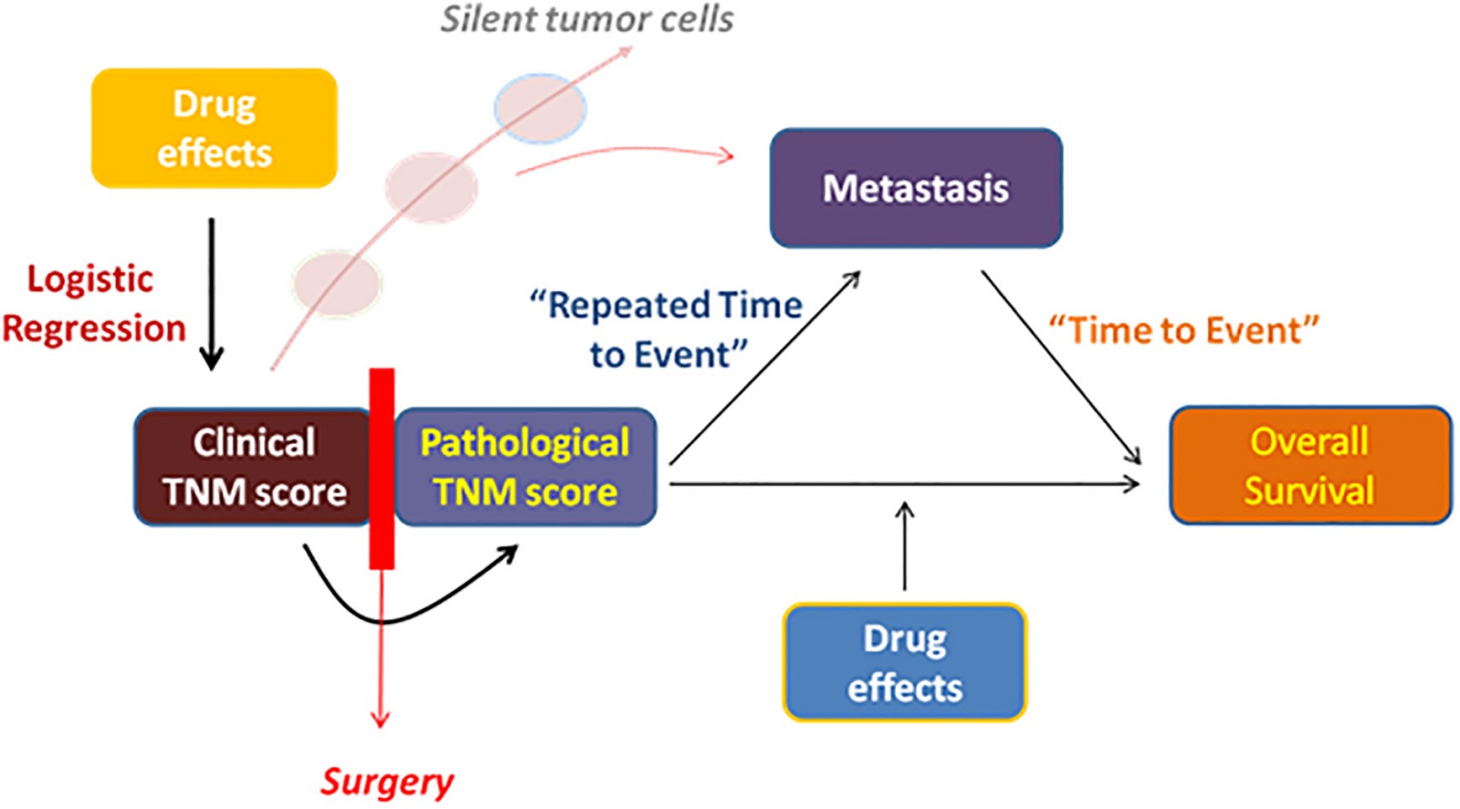
Schematic diagram of the workflow followed on the modeling exercise.

Two types of response data were analyzed and linked together through the computational modeling framework, whose graphical representation is depicted in Fig 1. (i) Tumor size data, were categorized based on the following criteria: 0–partial response, 1– stable disease; 2– disease progression (Table 1), where it was assumed that all patients presented progression of the disease at the time of diagnosis, and (ii) progression free survival (PFS) and overall survival (OS) data.

**Table 1:** Response model and relation with TNM stage during neoadjuvant treatment.

DV	Response criteria	Relation with TNM stage
0	Partial response	Downstaging during neoadjuvant treatment
1	Stable disease	No change during neoadjuvant treatment
2	Disease progression	Upstaging during neoadjuvant treatment

Tumor size data were treated as ordered categorical variables, and PFS and OS as time to event variables. Correlation between clinical and pathologic response was calculated by Spearman correlation coefficients to quantify bivariate correlations.

Once the model for tumor size data was established, the survival model was developed using predicted tumor size metrics as covariates of the hazard (see below).

#### Tumor response model

The response to preoperative therapy was analyzed as ordered categorical response variables, using logistic regression. The probability [P(Y_ij_)] of expressing a certain stage (Y) ≥ *m* (being *m* = 0, 1 or 2) in a patient i, on visit j (being j diagnosis, during treatment, post-treatment or surgery), is given by the following expression:

$$P_{Yij}=\frac{e^{L}}{1+\;e^{L}}$$ (1)

Patient’s’ response was modeled as a conditional probability P(Y_ij_ = m/η_i_), being η_i_ a random variable which corresponds to the disagreement between the median population value and the individual value. It is assumed that the combination of all individual η_i_ values follows a normal distribution, with a mean equal to 0 and a standard deviation to ω^2^. Finally, the probability of presenting a certain stage (Y) = *m*[P(Y_ij_ = *m*/ η_i_)] is:

$$P(Y_{ij}=\frac{m}{\eta_{i}})=P\;(Y_{ij}\geq \frac{m}{\eta_{i}})-\;P(Y_{ij}\geq \frac{m+1}{\eta_{i}})$$ (2)

The value of the probabilities expressed in Eqs 1 and 2 is comprised between 0 and 1. However, the logit value (L) can take any value between–and + infinite. Subsequently, the possible treatment effect, patient and tumor characteristics are incorporated on the logit structure as it follows:

$$L=f_{diagnosis}(m)+g(ChT)+\;h(CRT)+\;i(covariates)+\;\eta_{i}$$ (3)

ChT and CRT refer to chemotherapy and chemo-radiation treatment, respectively. The description of the stages at the time of diagnosis is described by f_diagnosis (m) and is expressed as, ∑_(k=1)_^m^ = ß_k_ where β_k_ (k = 1, …, m) are the parameters defining the diagnosis probabilities of having an ≥ m stage at diagnosis.

As for g(), h() and i(), refer to chemotherapy, chemoradiation and tumor-related characteristics, respectively. An example is given on the following equations:

$$g(CT)=\theta_{DOX}\times DOX+\;\theta_{FOLFOX}\times FOLFOX$$ (4)

$$h(CRT)=\theta_{RDT}\times CRT$$ (5)

$$i(Covariates)=\theta_{HER2}\times HER2+\;\theta_{LINITIS}\times LINITIS$$ (6)

In Eqs 4 and 5, θ_DOX_, θ_FOLFOX_ and θ_RDT_ correspond to the parameters that influence the probability of response to treatments with Docetaxel, FOLFOX and radiotherapy, respectively; θ_HER2_ and θ_LINITIS_ on Eq 6 represent the effect of HER2 and linitis plastica over the probability of response. During the modeling exercise, the statistical significance of g(), h() and i() was assessed.

The contribution of the Markov elements was also analyzed. Markov elements make the prediction of an observation dependent on the value of the previous observation, assuming that at the time of diagnosis all patients had progressive disease. In this case, the introduction of Markov elements into the ordered categorical response data had the advantage of being rather simple to apply [13, 14].

#### Survival model

A parametric ‘time to event’ approach was used to describe time-to-observation of PFS and OS. For the appearance of new lesions, time to event was set as the time of detection of a new lesion. For OS, time to event was defined as the time from diagnosis until death or last contact. Different distributions were explored for both types of data, such as exponential, Weibull or Gompertz models.

During the analysis, the effect of demographic, treatment and tumor-related characteristics on OS and PFS was evaluated. In order to build a time to event model, a hazard function was established, with the only constrain of being non-negative. The survival function (S) is expressed as: S(t) = e ^(-HZ)^ being HZ the accumulated value of the hazard function.

Since one of the aims of the study was linking treatment response to OS, we performed an exhaustive analysis in order to determine the covariates most likely related to OS, including age, gender, ECOG performance status, tumor location, tumor histological grading, Lauren classification, baseline T and N stages, type of neoadjuvant therapy (ChT vs CRT) and histopathologic response.

#### Model evaluation

The model was evaluated by performing 500 simulations of datasets within the same clinical study design as the ones available for the analysis. The 2.5^th^, 50^th^, and 97.5^th^ percentiles of the simulated observations in each dataset were computed for all time intervals and graphically represented along with the probability of response profile observed, visually evaluating the model. Parameter precision was further evaluated performing 200 non-parametric bootstrap analyses using Pearl Speaks NONMEM, listing the 2.5^th^, 50^th^ and 97.5^th^ percentiles of each parameter distribution.

## Results

### Patient characteristics

A total of 115 patients were included in the analysis. Eighty one patients (70.4%) were male. The median age at the time of diagnosis was 62 years old (range 36–83). EUS stage was cT3 in 75 patients (65.2%), cT4 in 30 patients (26.9%) and cN+ in 83 patients (72.2%). Primary tumor was GC in seventy-nine patients (69.6%) and GEJC in 36 (30.4%). Fifty (43.5%) and 65 56.5%) patients were treated with ChT and CRT, respectively. A complete pathologic response was achieved by 17 patients (14.8%). Patient and tumor characteristics are shown on Table 2.

**Table 2:** Patients’ characteristics.

Characteristic	Patients (%)
Age (median, range)	62 (31–83)
Gender	
Male	81 (70.4%)
Female	34 (29.6%)
ECOG performance status	
0	6 (5.2%)
1	109 (94.8%)
Tumor	
Gastric	79 (69.6%)
Gastroesophageal Junction	36 (30.4%)
Location	
Cardias	36 (30.4%)
Antrum	41 (35.7%)
Body	36 (31.3%)
Pylorus	2 (1.7%)
EUS-T stage	
cT2	10 (8.7%)
cT3	75 (65.2%)
cT4a	25 (21.7%)
cT4b	5 (4.3%)
EUS-N stage	
cN0	32 (27.8%)
cN+	83 (72.2%)
cTNM stage	
cII	35 (30.4%)
cIII	80 (69.6%)
Histologic grade	
Well differentiated	4 (3.5%)
Moderately differentiated	47 (40.9%)
Poorly differentiated	64 (55.6%)
Linitis plastica	
Yes	28 (24.3%)
No	87 (75.7%)
Lauren Histologic classification	
Diffuse	62 (53.9%)
Intestinal	53 (46.1%)
Neoadjuvant strategy	
CT	50 (43.5%)
CRT	65 (56.5%)
Surgery	
Total gastrectomy	61 (53.1%)
Subtotal gastrectomy	35 (30.4%)
Ivor-Lewis esophagectomy	19 (16.5%)
R0 resection	107 (93%)
Pathologic T classification	
ypT0	17 (14.8%)
ypT1	11 (9.6%)
ypT2	26 (22.6%)
ypT3	50 (43.5%)
ypT4a	8 (7%)
ypT4b	3 (2.6%)
Pathologic N classification	
ypN0	70 (60.9%)
ypN1	26 (22.6%)
ypN2	8 (7%)
ypN3a	10 (8.7%)
ypN3b	1 (0.9%)

### Tumor size model

Patients achieved a significant clinical response during treatment (p<0.01). This benefit was not related to the type of preoperative therapy (ChT or CRT) nor to the administered dose intensity (p>0.05). On the other hand, radiological response at each visit was significantly associated to the response categories measured in the previous visit (p<0.01). Such behavior was modeled using first order Markov chains. Therefore the selected structure for g(ChT) + h(CRT) in Eq 3 was reduced to θ_TRT,PVR_, with a value of 0 at diagnosis, or the corresponding model estimates depending on the response value measured at the previous visit (PVR, 0, 1, o 2, at visits 2 and 3). Table 3 lists the parameter estimates form the selected model. Estimated values for θ_TRT,PVR = 0–2_ suggest that the likelihood of achieving a response was dependent on the response achieved in the previous visit. The probability of achieving a partial response was 29.1%, 84.7% and 5.9% if the patient had previously presented a partial response, stable disease or disease progression, respectively.

**Table 3:** Population parameter estimates of response and survival models.

	Parameter	Estimate (RSE%)	2.5^th^-97.5^th^
**RESPONSE**	Clinical Baseline logit 1	19.7 (3%)	17.4–29.1
	Pathological Baseline logit 1	18.6 (3%)	16.6–27.6
	Clinical Baseline logit 2	-4.62 (13%)	(-15.3)–(-3.8)
	Pathological Baseline logit 2	-1.04 (19%)	(-1.6 –(-0.7)
	Markov component–Partial Response	18.1 (3%)	15.7–27.1
	Markov component—Stable Disease	19.4 (2%)	17.3–28.5
	Markov component–Disease Progression	18.8 (3%)	16.5–28.0
**OS**	Baseline	0.0001 (83%)	4·10^−5^–1·10^−3^
	Metastasis	3.43 (21%)	1.73–5.13
	AJCC	0.0001 (75%)	5.3·10^−5^–2.8·10^−3^
	Linitis Plastica	0.0002 (81%)	1·10^−5^–5·10^−4^

Clinical (EUS, CT-scan) and pathological stages (surgical specimen) correlated poorly with each other for both T (R = 0.32) and N (R = 0.19) classification. Correlation between the clinical response categories and the histopathological response was also low (R = -0.29). The parameter estimate of the probability of presenting a clinical stable disease was 19.9, while the probability of presenting a pathologic stable disease was 18.9. In the case of disease progression, the parameter estimates were -4.62 and -1.02 for clinical and pathological assessment, respectively, highlighting the differences between clinical and pathological determinations.

**Fig 2:**
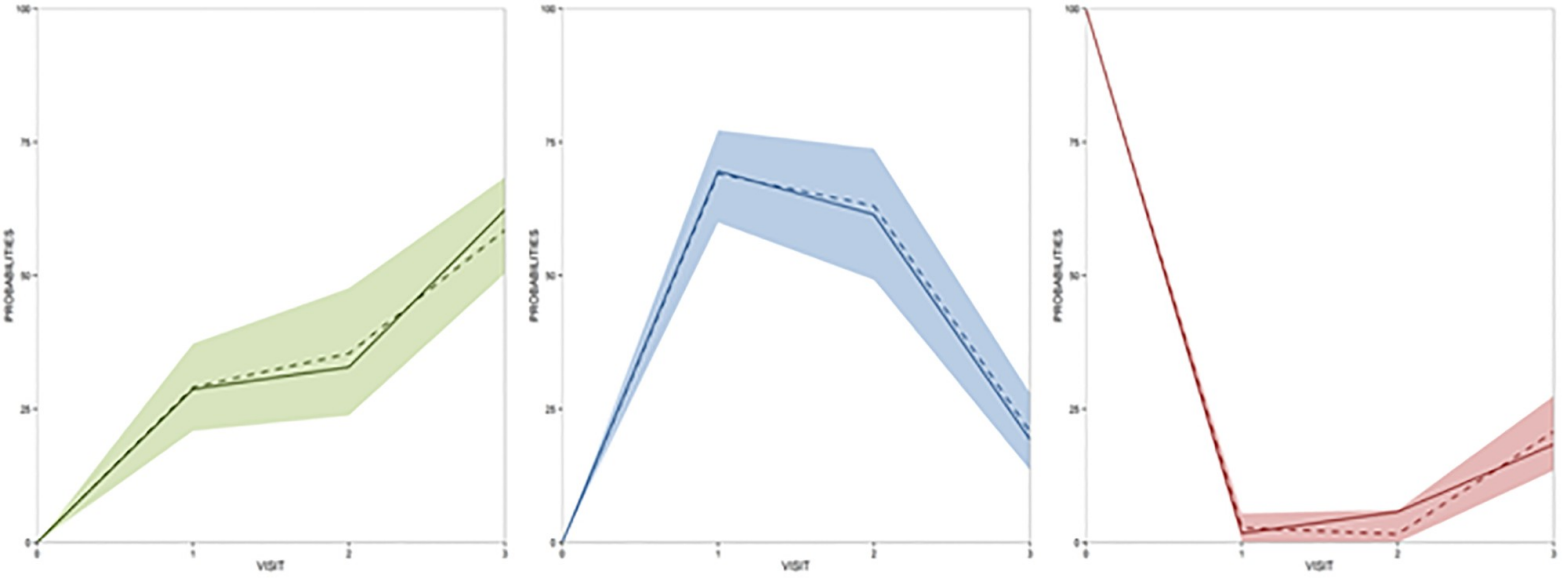
Results of the visual predictive checks of the model after 500 simulated profiles for the probability of response. Partial response in green (A), stable disease in blue (B), or progressive disease in red (C). Solid lines represent the observed raw data. Dotted lines correspond to the median of the simulated profiles.

Results from the model evaluation, including observed probabilities, are described by the selected model (Fig 2). All model parameters were precisely estimated, with relative standard errors below 20%, indicating good model performance (Table 3).

### Progression free and overall survival model

The exponential model provided a better description of the hazard rate of OS than other model distributions, such as a constant hazard, or a Weibull distribution model (p<0.01). Among the covariates effects explored, appearance of metastasis (p>0.05), linitis plastica (p<0.001) and TNM stage at diagnosis (p<0.05) showed an impact on OS. When the type and intensity of neoadjuvant treatment or the histopathological response were incorporated into the model, a statistically significant impact rate was not observed (p>0.05). The structure of the selected model was an exponential survival model, with covariates affecting the baseline hazard, and thus modifying the cumulative value of the hazard function.

**Fig 3:**
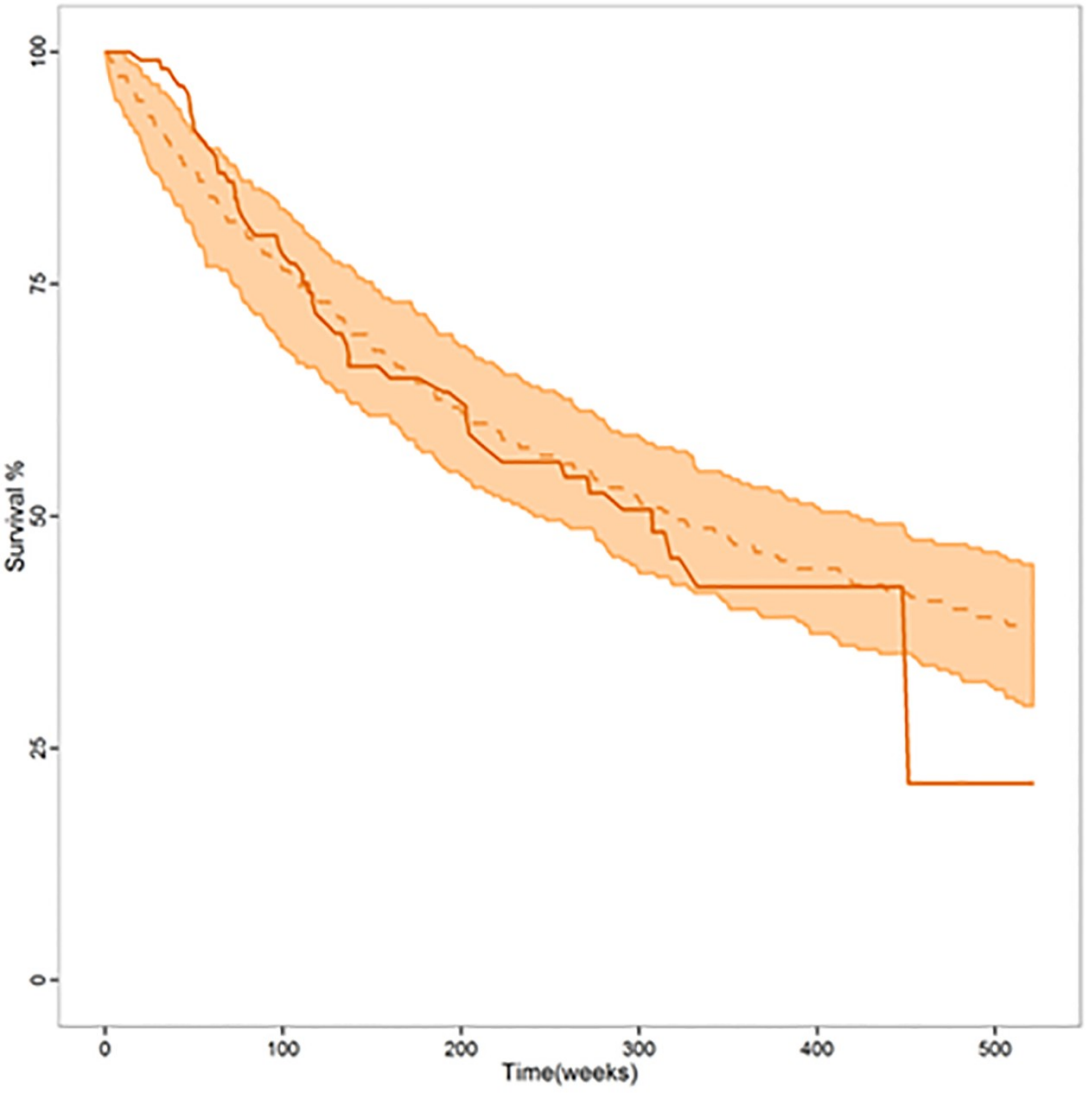
Results of the visual predictive check as a Kaplan-Meier curve after 500 simulations. Orange solid lines represent the observed probability of OS. Orange dotted lines represent the median simulated OS probability. Orange shaded areas represent the 95% prediction intervals of 500 simulated datasets.

**Fig 4:**
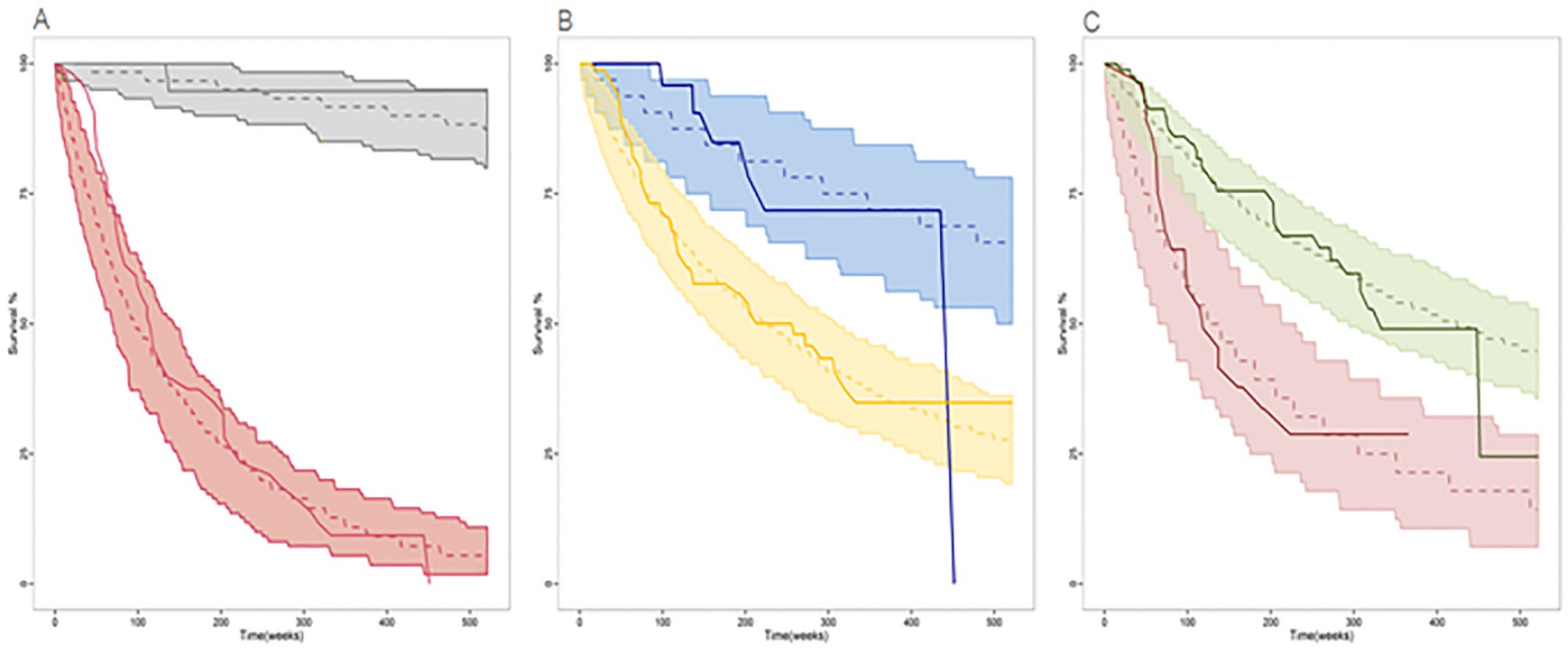
Results of the visual predictive checks as Kaplan-Meier curves after 500 simulations showing the probability of survival. The probability of OS (A) is depicted depending on the appearance (red) or absence (grey) of metastasis. The TNM stage at diagnosis (B) is categorized as <3 (blue) or ≥3 (yellow) to show the effect on OS probability. The probability of OS (C) is shown for patients with linitis (red) or without (green). Solid lines represent the observed OS probability; dotted lines represent the median simulated OS probability and shaded areas represent the 95% confidence intervals of 500 simulated datasets.

Table 3 lists the estimates of model parameters integrating the hazard rate and the corresponding relative standard errors. Figs 3 and 4 show that the model developed for OS provides a very good description of the probability of survival profiles for the entire patient population, also when stratified by the selected covariates.

Metastasis development was associated to a higher risk of death, as well as to clinical stage III and to the presence of linitis plastica at diagnosis (Fig 4). The 8-year survival for patients who develop metastasis was 57.3%, compared to an 8-year OS of 100% (Hazard Ratio [HR] = 33, p = 1.6·10^−6^) in patients without metastases. Similarly, the 8-year survival in patients with and without linitis plastica was 56.7% and 85%, respectively (HR = 2.9, p = 0.0014). Patients with a baseline TNM stage III had a significantly shorter 8-year survival compared to patients with a lower TNM stage (71% vs 97%, HR = 2.8, p = 0.032).

## Discussion

In recent years, several neoadjuvant strategies in patients with locally advanced gastric and gastroesophageal adenocarcinoma have gained acceptance, due to promising data in terms of R0 resection rates, histopathological responses, therapy compliance and early treatment of micrometastatic disease [4–9]. Nevertheless, up to 15–20% of the patients develop interval progression during the preoperative therapy, thus precluding a potentially curative surgery [4, 7]. In addition, the toxicity profile is an important issue especially with the use of preoperative CRT, with up to 30% of patients requiring hospital admission due to grade 3–4 toxicity [15]. Even in patients undergoing a complete neoadjuvant program, relapse rate is as high as 50% [4–8]. Finally, the subset of patients more likely to benefit from a more intensive approach, in terms of increasing the number of chemotherapy cycles or incorporating the use of preoperative radiation remains undetermined. Pathologic response and tumor downstaging are usually considered as surrogate markers of individual efficacy of neoadjuvant therapies. Several studies have suggested that pathologic stage (ypTNM) and especially nodal status (ypN0) strongly dictate prognosis, rather than clinical stage at diagnosis (cTNM) [16–18]. However, ypTNM stage is only available once the patient has undergone surgery and therefore cannot be employed as a baseline predictive marker. In addition, although several molecular biomarkers and CTCs dynamic changes have been associated with therapeutic response in this disease, none of them has so far been prospectively validated [18–22]. Early identification of the degree of response in an individual patient would lead to either an intensification of the neoadjuvant strategy in responding patients or to consider an early surgical approach in the absence of clinical benefit.

In the present work, we intended to develop a mechanistic PK/PD model in patients with CG and GEJC treated with neoadjuvant ChT with or without CRT and subsequent surgery. Mechanistic PK/PD models can describe individual variability in population level trends [23]. These models also provide an individual prediction using Bayesian methodology [11]. A mechanistic model in locally advanced GC able to accurately predict response to neoadjuvant therapy would be of interest in these patients. Interestingly, our model was capable of adequately describe the risk of presenting disease progression, stable disease and partial response. Although a response-based PK/PD model seemed appealing, our results showed that the present model was unable to predict the degree of pathologic response on an individual basis. It must be emphasized that the analysis was greatly influenced by the extremely low correlation between the clinical and pathologic stages (R = -0.3). Clinical stage is defined at diagnosis by CT-scan and endoscopic ultrasound. The accuracy of these pretreatment imaging is controversial, with a sensitivity and specificity in the range of 70–80%. Another limitation of these imaging techniques is their inability to discriminate between the presence of residual tumor and treatment-related changes as fibrosis or inflammation [24–26]. In a recent study that assessed the clinical response by a combination of CT-scan and EUS in GC patients a pathologic response was observed in 92% of patients who had a lack of clinical response, with a correlation between clinical and pathologic response of only 50% [27]. These results may suggest that the absence of clinical response rather than its presence might be a surrogate of neoadjuvant treatment efficacy. Functional imaging techniques such as PET-scan have also been evaluated in locally advanced gastric and gastroesophageal adenocarcinoma, mostly in retrospective trials with disappointing results, mainly due to a restricted avidity for FDG especially by signet cell in diffuse tumors [28, 29]. Other alternatives such as the combination of endoscopic and laparoscopic ultrasound have shown promising results as post-treatment staging tests, although their results should be validated in prospective studies [24, 30].

Another limitation of our analysis is the small number of observations available for each patient in comparison to previously published mechanistic PK/PD models [31, 32]. Individuals from our series had a maximum of three observations (CT or EUS) during the neoadjuvant treatment, which might have precluded an accurate evaluation of therapy efficacy as well as a quantification of inter-patient variability. Moreover, although some studies have suggested that the addition of neoadjuvant radiation increases the likelihood of achieving a favorable pathologic response, the inclusion of CRT into the model was not a significant predictor of response [5, 8, 9]. An alternative proposed approach to predict clinical outcome in these patients is the use of a more reliable variable of the disease dynamics, such as tumor size changes during treatment, an easily attained variable related to treatment efficacy, or dynamic changes in serum tumor markers that, unfortunately, lack of specificity in this disease [33].

In order to identify predictive factors of tumor burden dynamics and response, the Markov component was included in the modeling exercise. The Markov component is a stochastic process that predicts the future state of the study variable depending upon the current state of that variable, despite the event sequence it was preceded by. A stochastic process can be defined as a random variable whose study is done under the foundations of probability theory. In this case, Markov component was used to study the change of TNM stage, which determines the probability of presenting a given category in a specific moment, considering the TNM stage in the previous measurement time. This modeling strategy was used as an alternative to continuous analysis of tumor size, due to limitations of the study such as insufficient number of observations and the lack of correlation between clinical and pathological staging, as discussed above. This analysis could identify Markov patterns of response as a prognostic factor for OS. Additionally, the presence of metastasis, linitis plastica or a TNM stage III at diagnosis were associated to a higher risk of death. Moreover, the survival parameter from our model estimated that patients who developed metastasis during the follow-up almost tripled the risk of death at 4 years after diagnosis. Consistent with previous reports, results from our model confirm disease recurrence as a prognostic factor for a decreased OS [4, 16].

Current available biomarkers are insufficient to adequately define GC patients’ prognosis [1]. Although genomic landscape of GC has been defined recently, integration of both genotype and phenotype remains an unmet need for GC patients. The use of mechanistic PK/PD models enables the identification of important covariates that determine response with the aim of personalizing treatment [11]. In this setting, there is an increasing interest in using PK/PD models as Bayesian priors, with the observed patient PD endpoint and covariate information being used to construct a posterior set of most likely individual model parameters to be used to predict or adjust future treatment [34].

## Conclusions

In conclusion, we aimed to develop a mechanistic PK/PD model able to predict response to a neoadjuvant approach including chemo and radiotherapy. The current model does not allow predicting the pattern of response in this subset of patients. The low correlation between clinical and pathologic stages, the inaccuracy of imaging techniques to properly evaluate response to neoadjuvant therapies and the lack of reliable serum tumor markers in this disease make this model unlikely to be a useful tool for precision medicine.

## Supporting information

S1 File Study’s underlying anonymized data set. (XLSX)
